# Protective Efficacy of BCG Vaccination in Calves Vaccinated at Different Ages

**DOI:** 10.3390/pathogens12060789

**Published:** 2023-05-31

**Authors:** Jayne C. Hope, Hamza Khalid, Michelle L. Thom, Chris J. Howard, Darren J. Shaw

**Affiliations:** 1 Division of Infection and Immunity, The Roslin Institute, and Royal (Dick) School of Veterinary Studies, The University of Edinburgh, Easter Bush, Midlothian EH25 9RG, UK; h.khalid-4@sms.ed.ac.uk (H.K.); darren.shaw@ed.ac.uk (D.J.S.); 2 Institute for Animal Health, Compton RG20 7NN, UK; michelle.thom@pirbright.ac.uk (M.L.T.); c.howard41@btinternet.com (C.J.H.); 3 The Pirbright Institute, Pirbright GU24 0NF, UK

**Keywords:** age, BCG, bovine tuberculosis, *Mycobacterium bovis*, vaccination

## Abstract

*Mycobacterium bovis*, the causative agent of bovine tuberculosis (bTB), is a globally prevalent pathogen with significant animal welfare, economic and public health impacts. In the UK, the control of bTB relies on detection via tuberculin skin tests with ancillary interferon gamma (IFN-γ) release assays, followed by culling infected animals. Vaccination with Bacille Calmette–Guérin (BCG) could be an important element of bTB control, and a number of studies have demonstrated its protective efficacy, particularly when young calves are vaccinated. Here, we compared immune responses and the protective efficacy of BCG in calves vaccinated within the first day of life and at three weeks of age. Significant protection from *M. bovis* infection was observed in BCG-vaccinated calves compared to non-vaccinated, age-matched controls. No significant differences were shown between calves vaccinated at one day and at three weeks of age when assessing the protective efficacy of BCG (measured as a reduction in lesions and bacterial burden). Antigen-specific IFN-γ levels were similar between the BCG-vaccinated groups, but significantly different from the non-vaccinated control animals. Antigen-specific IFN-γ expression post-BCG vaccination was correlated significantly with protection from *M. bovis* infection, whereas IFN-γ levels post-challenge correlated with pathology and bacterial burden. These results indicate that early-life vaccination with BCG could have a significant impact on *M. bovis* infection and, therefore, bTB incidence, and they demonstrate that age, at least within the first month of life, does not significantly impact the protective effect of vaccination.

## 1. Introduction

Bovine tuberculosis (bTB), caused by *Mycobacterium bovis*, is a globally prevalent, chronic infectious disease with significant consequences for animal trade, economics and welfare. A conservative estimate suggests that 50 million cattle are infected worldwide with *M. bovis*, with losses of 3 billion dollars per annum [1]. Over the past decade, the UK alone has spent over a billion pounds on the control of bTB [2], and the disease remains of significant impact. Currently, the diagnosis of bovine TB is carried out via a tuberculin skin test and ancillary blood tests that detect antigen-specific interferon gamma (IFN-γ) secretion; bTB-affected cattle are subsequently culled from affected herds [3]. The control of bTB is complicated by the presence of *M. bovis*-infected wildlife reservoirs, including the Eurasian badger [4]. It is clear that improved strategies are required to reduce the incidence of bTB, and the vaccination of cattle is considered as a key aspect of bTB control.

Currently, the only licensed vaccine against human TB is Bacille Calmette–Guérin (BCG), an attenuated form of *M. bovis*. In cattle, a wide range of studies of BCG efficacy, in both experimental and natural transmission settings [5,6,7,8,9,10,11], strongly indicate that although BCG does not provoke sterilising or highly protective immunity, it is effective enough to have a significant impact on the incidence of bTB. We, and others, have previously demonstrated the significant protective efficacy of neonatal BCG delivery in calves [7,8,12,13], whereas with advancing age, BCG’s efficacy and protection is reduced [14].

A recent review and meta-analysis of BCG vaccination efficacy across cattle studies [15], together with modelling approaches, showed strong evidence that BCG vaccination would positively impact bTB control. Although the overall protective effect of BCG was shown to be relatively low (approximately 25%), it was suggested that a BCG vaccine-based control strategy would be effective. This could be particularly beneficial in high-burden settings such as low- and middle-income countries (LMICs), where *M. bovis* impacts both cattle and human populations, and in countries such as the UK, where bTB is endemic in cattle herds. The implementation of BCG vaccination could limit spread of the disease, thereby enabling further development of alternative approaches to disease control, e.g., through the development of more sensitive diagnostic tests or novel vaccines [15,16].

In the UK, current field trials of BCG aim to provide further evidence for the use of vaccination as part of a suite of tools to improve the control of bovine TB. Because BCG is an attenuated form of *M. bovis*, vaccinated animals show responses against bovine tuberculin (PPD-B) because of T cell cross-reactivity; this prevents the accurate use of tuberculin skin tests and IFN-γ release assays [6,17]. This interference in specificity of diagnostic tests has hampered the deployment of BCG vaccination in the field. However, the use of a recently developed DIVA skin test (DST) [18,19,20] will enable the differentiation of *M. bovis*-infected from BCG-vaccinated animals, further facilitating the deployment of BCG in the field.

Here, we provide further evidence for the efficacy of BCG vaccination in young calves. Although one of our earlier studies utilised calves vaccinated with BCG within the first day of life [8], here, we extended a comparative analysis of calves aged up to 3 weeks of age, and we demonstrate no significant differences in vaccine-induced immunity between animals vaccinated at 1 day and at 3 weeks of age. This enables a more practical window of opportunity to immunize young calves with BCG while retaining significant vaccine protective efficacy against extensive *M. bovis* infection.

## 2. Materials and Methods

### 2.1. Animals

The cattle tested were British Holstein–Friesian calves (*Bos taurus*) bred at the Institute for Animal Health (IAH), Compton, Berkshire, UK. The calf numbers, sex and age at time of vaccination are detailed in Table 1. The IAH herd had been confirmed free from bovine TB for more than ten years at the time of the study. Animals were vaccinated with BCG when aged one day or three weeks of age (Table 1). Age-matched control animals were non-vaccinated.

Calves were assigned to groups according to availability, and groups of 4–5 calves were used. Between 5 and 6 months following vaccination, calves were transferred into a high-security, ACDP category 3 animal housing unit and challenged with *M. bovis* via the intranasal route. Twelve weeks following infection, tuberculin skin tests were performed. The animals were killed one week later, and post-mortem examinations were performed as described later. The experiments were carried out under a Home Office Project Licence and were approved by the local ethics committee according to the National UK guidelines.

### 2.2. Bacteria and Inoculation

BCG Pasteur (sourced from the Animal and Plant Health Agency, Weybridge, UK) was diluted from previously titrated frozen (−70 °C) stock grown in Middlebrook 7H9 broth containing 10% ADC supplement. Calves were vaccinated with approximately 1 × 10^6^ cfu subcutaneously (Table 1). For infection, *M. bovis* (strain AF 2122/97 [21]) was diluted 1/10 from frozen stock in 7H9 medium and incubated at 37 °C for 7 days. The optical density was measured, and the number of *M. bovis* colony-forming units was calculated. For intranasal inoculation, a short 10-cm cannula was fitted with a syringe adaptor at one end and pierced 8–10 times with a 21G’ needle in the last 2 cm at the opposite end, which had previously been sealed. The cannula was inserted into the back of the nasopharyngeal area of the animals, perforated end first, and 2 mL of 7H9 containing *M. bovis* was sprayed into the calf. Before removal from the calf, the cannula was rinsed with an additional 2 mL of 7H9 medium. The dose of *M. bovis* was determined retrospectively via titration on modified 7H11 agar (Table 1).

### 2.3. Post-Mortem Examination and Bacteriology

Lymph nodes of the head (retropharyngeal, sub-mandibular and parotid) and thorax (mediastinal and four associated bronchial lymph nodes), the tonsils, nasal and tracheal mucosa and the seven pulmonary lobes were examined for gross lesions following the cutting of 0.5 to 1 cm slices. Macroscopic lesions were scored as previously described [22]. Tissues were fixed in 10% formal saline and processed for histological examination following staining with Haematoxylin and Eosin. Tissues with typical lesions of TB evident under microscopy but with no gross lesions were given a score of 1 in the overall pathology comparison. Samples from the same tissues were frozen at −70 °C for subsequent bacteriological examination via titration of tissue homogenates on modified 7H11 agar [23]. Tissues were homogenized with a pestle and mortar in water containing Tween 80, and 1-mL volumes were plated in duplicate on 7H11 agar. After 3 weeks of culturing at 37 °C, colony-forming units (cfu) were counted, and the average cfu was calculated per tissue. The cfu counts for all of the tissues within each individual animal were added together to calculate the total cfu.

### 2.4. Antigens

Purified protein derivatives from *M. avium* (PPD-A) and *M. bovis* (PPD-B) as well as *M. bovis*-specific proteins Early Secreted Antigenic Target-6 (ESAT-6) and Culture Filtrate Protein-10 (CFP-10) were obtained from the Tuberculin production unit at Veterinary Laboratories Agency (VLA), Weybridge.

### 2.5. Tuberculin Skin Tests

The single comparative intradermal tuberculin test with avian and mammalian PPD (PPD-A and PPD-B, respectively) was done via intra-dermal inoculation of 0.1 mL of PPD-A and PPD-B, and reactions were read 72 h later. Results were recorded as increases in skin thickness at 72 h compared to thickness pre-injection, and they were interpreted according to the standard protocol (European Communities Commission regulation number 1226/2002) [24].

### 2.6. Immunological Assays

All calves’ blood was sampled via venepuncture of the jugular vein at bi-weekly intervals throughout the study. Blood was collected into heparin (10 U/mL). To measure IFN-γ, 4 mL of blood was incubated at 37 °C for 24 h with antigens (PPD-A and PPD-B at a final concentration of 10 μg per mL each; ESAT-6 and CFP-10 at a final concentration of 5 μg/mL each). Antigens were diluted in RPMI with 50 μg/mL gentamicin (medium) or in medium alone. The supernatant was removed after centrifugation and stored at −20 °C until assayed. Antigen-specific IFN-γ was determined up to 12 weeks post-BCG and up to 12 weeks post-*M. bovis* challenge. IFN-γ was assayed via enzyme linked immunosorbent assay (ELISA) as described previously and by using recombinant bovine IFN-γ as a standard [25]. Each sample assayed was measured in duplicate via ELISA; the variability between samples was less than 5%.

### 2.7. Statistical Analysis

All statistical analyses were carried out in R (v4.2.0 © 2022 The R Foundation for Statistical Computing) from within RStudio (2022.07.1 © 2023-2022 RStudio, PBC). In all cases, a *p*-value of <0.05 was taken to indicate statistical significance.

Differences in bacterial and lesion scores taken from organs at post-mortem at 12 weeks pre- and post-challenge between calf groups were assessed via Kruskal–Wallis, and if overall differences were observed, pairwise differences between groups were assessed using the Wilcoxon rank sum test. Correlations between lesion scores, bacterial scores and skin test results were compared with Spearman rank correlations.

To calculate the PPD-B-specific IFN-γ values, the values obtained from Medium stimulation were first subtracted from the raw PPD-A and PPD-B values. This resulted in some negative corrected PPD-A and PPD-B values. Therefore, the absolute value of the minimum corrected PPD-A and PPD-B were added to all corrected PPD-A and PPD-B, respectively, to generate corrected positive PPD-A and PPD-B values. PPD-B-specific IFN-γ values were then obtained by calculating the difference between these two. Finally, prior to analysis, PPD-B-specific IFN-γ data were log_10_ transformed in order for the residuals to be normally distributed. For both ESAT-6- and CFP-10-specific IFN-γ values, Medium values were again subtracted from raw ESAT-6 and CFP-10 values, the absolutes of the minimum corrected values of ESAT-6 and CFP-10 were added to the corrected values ESAT-6 and CFP-10, respectively, to produce the specific IFN-γ data, and log_10_ transformation was undertaken in order for the residuals to be normally distributed.

For the analysis of differences in PPD-B-, ESAT-6- and CFP-10-specific IFN-γ values between calf groups during the 12 weeks pre- and post-challenge, linear mixed-effect models were carried out using the *lme4 (1.1-30)* [26] and *sjPlot (v2.8.10)* packages. Which calves were sampled at any one time point were entered as the random effect, which week the sample had come from was a covariate, and which group the calf was from was a fixed effect. Differences in changes with time were assessed by considering the interactions between weeks and groups.

Correlations between (a) PPD-B-, ESAT-6- and CFP-10-specific IFN-γ values and (b) lesion and bacterial scores were also assessed with Spearman rank correlations. Cumulative PPD-B-, ESAT-6- and CFP-10-specific IFN-γ values were also calculated and compared against lesion and bacterial scores.

## 3. Results

### 3.1. Protection Induced by BCG Vaccination Is Not Significantly Different in Calves Vaccinated at 1 Day or 3 Weeks of Age

Calves were vaccinated with BCG when aged 1 day or 3 weeks of age. Age-matched controls were non-vaccinated. Approximately 5–6 months later, all calves were challenged via the intranasal route with *M. bovis*. The protective effect of BCG vaccination against disease was assessed via post-mortem examinations performed 13 weeks post-challenge. None of the animals showed clinical signs of disease during the infectious challenge period (anorexia, poor responsiveness, rapid or difficult breathing and coughing).

**Figure 1:**
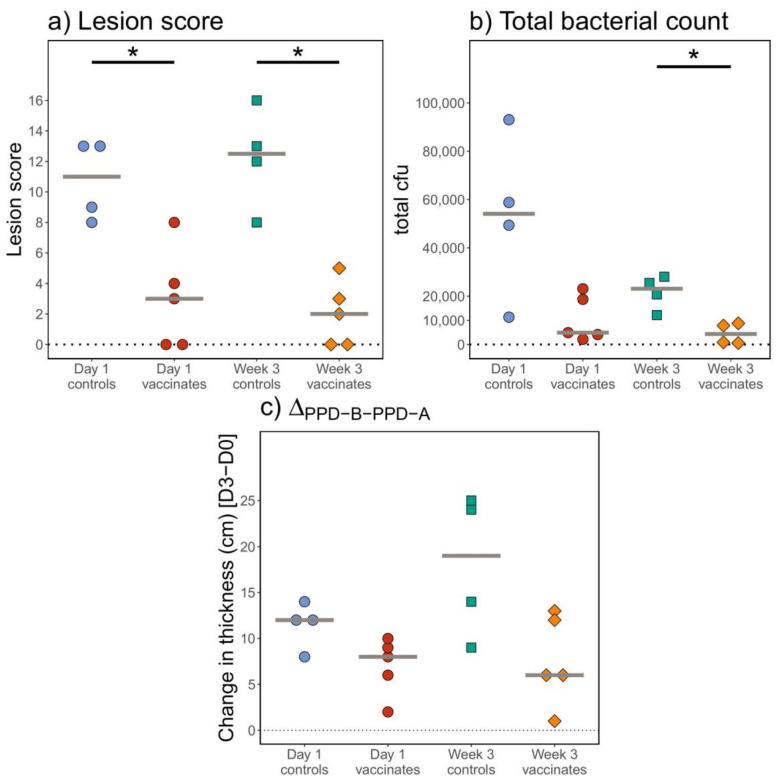
Protection conferred via BCG vaccination administered at different ages. Animals were vaccinated with BCG at day 1 of life (red) or at the age of 3 weeks (orange) and were challenged with *M. bovis* 5–6 months later. Age-matched controls are shown for both groups in blue (for day 1) and green (for week 3) (*x*-axis). (**a**) Total lesion scores at necropsy 13 weeks post-*M. bovis* challenge (*y*-axis). (**b**) Total *M. bovis* bacterial counts in colony-forming units (cfu) (*y*-axis) in tissues taken post-mortem from BCG-vaccinated and control animals. (**c**) Tuberculin skin test (_PPD-B–PPD-A_) responses are depicted on the *y*-axis with PPD-B-specific changes in skin thickness at day 3 minus the thickness at day 0. * *p*-value < 0.05.

We first assessed protection as a reduction in the lesion score, which reflects the size, distribution and number of tuberculous lesions in the head and respiratory tract. Lesions typical of TB were observed in all of the non-vaccinated control animals. Although the extent of these lesions varied between animals, as reflected by the lesion scores, no significant differences between the non-vaccinated control groups were observed (*p* = 0.766; Figure 1a). In each of the control groups, lesions were primarily restricted to the head and upper respiratory tract-associated lymph nodes. Only one animal (#629) had lesions in the diaphragmatic lung (data not shown).

In contrast, in animals at either age, BCG vaccination significantly reduced lesion scores compared to the age-matched, non-vaccinated controls (*p* < 0.026; Figure 1a). This was evidenced not only by a reduction in the number, but also the size of TB lesions in the vaccinated animals (data not shown). No significant differences were seen between the vaccinated groups, indicating that the age of vaccination did not impact the protective efficacy of BCG (*p* = 0.746).

### 3.2. BCG Vaccination of Calves Reduces Bacterial Burden in Calves

The bacteriological examination of tissues indicated that *M. bovis* was present in the majority of tissues with gross lesions across the groups. No significant differences were observed between the control non-vaccinated groups (*p* = 0.343). In vaccinated animals, *M. bovis* was isolated from a number of tissues (16/90 tissues) where gross or microscopic lesions were not observed. However, fewer bacteria were isolated from the tissues taken from calves in the BCG-vaccinated groups compared to their respective age-matched control calves (Figure 1b), with the difference being significant when comparing animals vaccinated at 3 weeks of age (*p* = 0.029). No differences were observed when comparing animals vaccinated at 1 day and at 3 weeks of age (*p* = 0.413), suggesting that the age of vaccination did not impact the capacity of BCG vaccination to reduce bacterial burden in the tissues examined. The overall bacterial burden was strongly correlated with the lesion score when all animals were assessed (*p* < 0.001, data not shown).

### 3.3. Skin Test Reactivity Did Not Differ between BCG-Vaccinated and Control Calves Following M. bovis Challenge

Reactions to PPD in the skin test were observed in all animals 12 weeks after challenge with *M. bovis* irrespective of BCG vaccination status (Figure 1c). Visible local clinical signs and increases in comparative skin thickness (skin test thickness response to PPD-B minus PPD-A) ranging from 2–25 mm were recorded. Low responses to PPD-A were noted, and all animals would be classified as reactors according to the standard interpretation of the skin test. There were no significant differences between any of the groups of animals (*p* = 0.064).

### 3.4. BCG Induced Similar Kinetics and Intensity of Antigen-Specific IFN-γ Responses in Calves Vaccinated at 1 Day or 3 Weeks of Age

**Figure 2:**
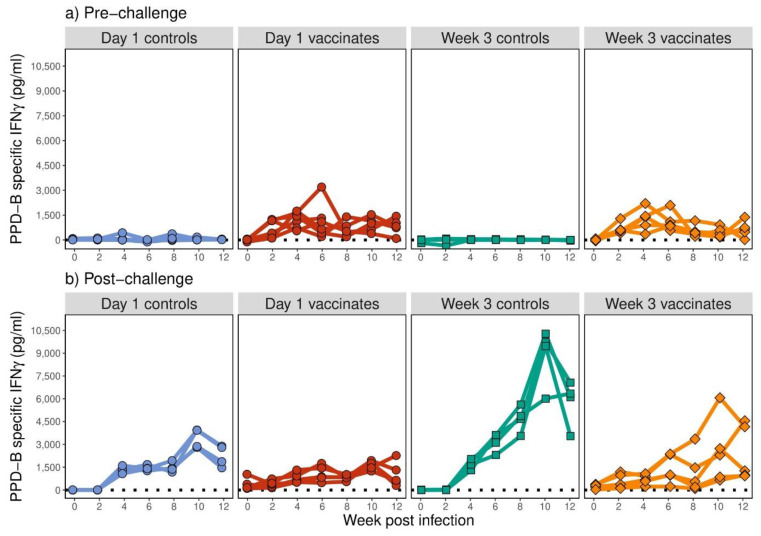
IFN-γ secreted in whole blood supernatants following 24 h of stimulation with bovine tuberculin (PPD-B) expressed in picograms per millilitre (pg/mL, *y*-axis). Calves were vaccinated at day 0 (red) and week 3 (orange) of life with BCG Pasteur. Age-matched control animals (blue for day 0 and green for week 3) were non-vaccinated. Immune responses were monitored for 12 weeks post-BCG vaccination (**a**) and post-*M. bovis* challenge (**b**).

Immune responses in the form of PPD-B-specific IFN-γ were determined in all calves up to 12 weeks post-vaccination and post-*M. bovis* challenge (Figure 2). None of the animals showed elevated antigen-specific IFN-γ prior to vaccination. Vaccination with BCG induced significant increases in PPD-B-specific IFN-γ secretion that were evident as early as 2 weeks post-vaccination (Figure 2a). During the pre-challenge period, no significant differences were observed between the groups of animals vaccinated with BCG (*p* = 0.964). BCG vaccination induced significant PPD-B-specific IFN-γ compared to their relevant age-matched control groups (Figure 2a; *p* < 0.001). Little or no IFN-γ was observed in the non-vaccinated control animals, and the groups did not differ significantly from each other (*p* = 0.272).

Approximately 5–6 months post-BCG vaccination, all calves were challenged with *M. bovis*. No significant differences were noted between the vaccinated groups post-challenge, either overall (*p* = 0.683) or in changes in PPD-B-specific IFN-γ with time (*p* = 0.182; Figure 2b). No significant differences in post-challenge PPD-B-specific IFN-γ compared to pre-challenge was observed in day 1 vaccinates (*p* = 0.947); however, increased PPD-B-specific IFN-γ was observed in week 3 vaccinates post-challenge compared to pre-challenge (*p* = 0.012).

In the non-vaccinated animals, challenge with *M. bovis* also stimulated an increase in PPD-B-specific IFN-γ, with significant differences observed from week 4 post-challenge compared to pre-challenge (*p* < 0.001; Figure 2b). Furthermore, PPD-B-specific IFN-γ in the week 3 non-vaccinated controls increased to higher levels compared to day 1 non-vaccinated controls (*p* = 0.007). In both of the BCG-vaccinated, *M. bovis* challenged groups, PPD-B-specific IFN-γ did not increase as much as the age-matched non-vaccinated and challenged animals (Figure 2b; *p* < 0.001).

**Figure 3:**
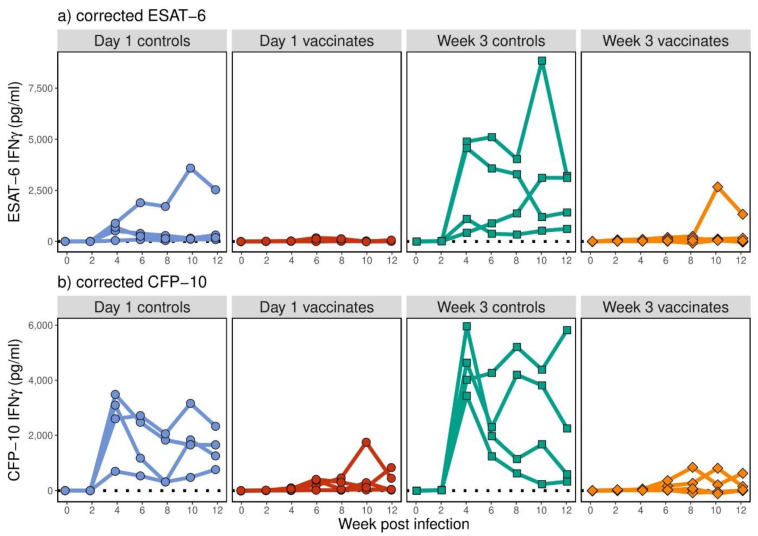
IFN-γ secreted in whole blood supernatants following 24 h of stimulation with ESAT-6 (**a**) and CFP-10 (**b**) expressed in pictograms per millilitre (pg/mL, *y*-axis). Calves were vaccinated at day 0 (red) and week 3 (orange) of life with BCG Pasteur. Age-matched control animals (blue for day 0 and green for week 3) were non-vaccinated. Shown are ESAT-6- and CFP-10-specific IFN-γ levels monitored for 12 weeks post-*M. bovis* challenge.

No ESAT-6- or CFP-10-specific IFN-γ was detected prior to challenge with *M. bovis* in any of the animals across all four animal groups (data not shown). Post-*M. bovis* challenge, the ESAT-6- (Figure 3a) and CFP-10-specific (Figure 3b) IFN-γ responses were highly variable, with some animals demonstrating high-level secretion of antigen-specific IFN-γ, and others showing much lower levels (Figure 3). Overall, the non-vaccinated control groups did show an increase in both ESAT-6- and CFP-10-specific IFN-γ levels post-challenge (*p* < 0.001), but no differences were observed between the two non-vaccinated groups (*p* > 0.169). No significant overall changes in ESAT-6-specific IFN-γ in the BCG-vaccinated groups and CFP-10-specific IFN-γ levels in the week 3 vaccinated group were observed with time (*p* > 0.094). There was a significant increase in CFP-10-specific IFN-γ levels in the day 1 vaccinated group (*p* < 0.001), though the mean increase by week 12 was very low. ESAT-6- and CFP-10-specific IFN-γ levels did not significantly differ between the two BCG-vaccinated groups (*p* > 0.089), but in both the day 1 and week 3 vaccinated groups, ESAT-6- and CFP-10-specific IFN-γ levels were significantly lower than their age-matched, non-vaccinated groups (*p* < 0.012).

### 3.5. Antigen-Specific IFN-γ Expression Post-Vaccination Correlated with Protection Whereas Expression Post-Challenge Correlated with Pathology and Bacterial Burden

**Figure 4:**
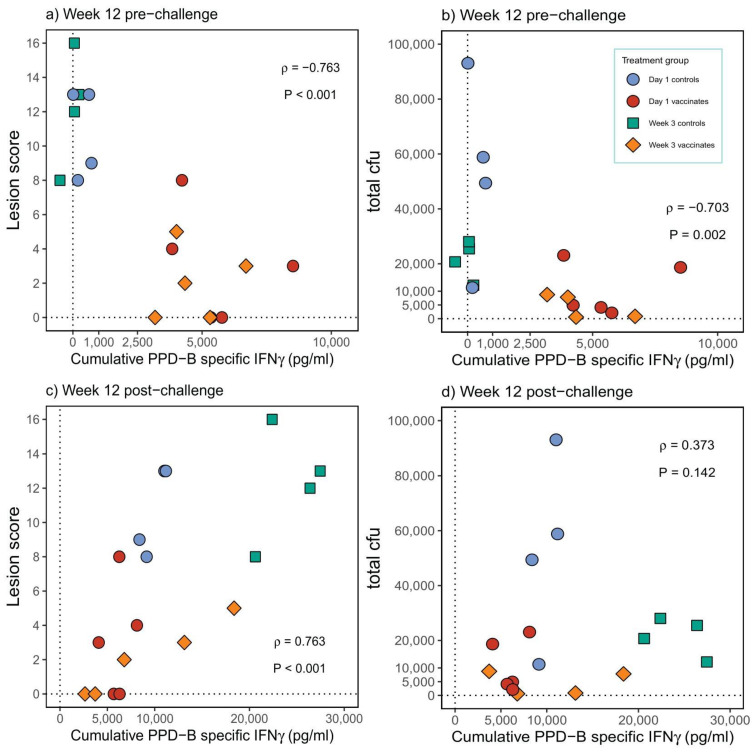
Correlation of cumulative PPD-B-specific IFN-γ (pg/mL) with total lesion scores post-vaccination (**a**) and post-*M. bovis* challenge (**c**) and with total bacterial count (cfu) post-vaccination (**b**) and post-*M. bovis* challenge (**d**).

Correlations between the total PPD-B-specific IFN-γ secreted in the post-vaccination (Figure 4a,b) and the total PPD-B-specific IFN-γ secreted in the post-*M. bovis* challenge period (Figure 4c,d) in terms of pathology (lesion scores) and bacterial burden (cfu count) were assessed. The total level of PPD-B-specific IFN-γ measured pre-challenge was found to significantly negatively correlate with lesion score and bacterial burden, indicating that this is a measure of protective immunity (Figure 4a,b; *p* > −0.702, *p* < 0.003). By contrast, the total amount of PPD-B-specific IFN-γ measured following *M. bovis* challenge was significantly positively correlated with pathology (Figure 4c, *p* < 0.001) but not with cfu count (Figure 4d, *p* = 0.142).

## 4. Discussion

Previous studies have reported the protective effect of neonatal BCG vaccination in calves against *M. bovis* challenge at various time points with respect to BCG administration (e.g., week 0, 3, 6 of age) [8,27]. It has also been reported that BCG efficacy is reduced in adults compared to young calves [14,28]. Here, we extended our studies of BCG efficacy in neonatal calves, comparing the response in calves vaccinated within the first day of life and at three weeks of age. In humans, vaccination of newborns within the first 24 h of life with BCG protects infants and young children against disseminated forms of tuberculosis (TB) and, to some extent, against pulmonary TB [29]. We previously showed the significant protective efficacy of BCG vaccination of one-day-old calves [8]. However, the administration of BCG to calves within the first hours of life may not be practical depending on cattle herd management approaches, and therefore, we compared the BCG vaccine responses of newborns to those of three-week-old calves. No significant differences in the protective efficacy of BCG vaccination were observed when comparing animals of different ages, and significant protection was observed compared to non-vaccinated, age-matched control calves. As reported previously for both experimental and natural *M. bovis* infection, BCG did not induce sterilising, fully protective immunity. However, protective efficacy was shown to be greater than 70% with respect to a reduction in both bacterial counts and lesion scores in BCG-vaccinated calves. In reviewing studies of BCG vaccination in cattle, Srinivasan et al. [15] determined that even with a protective efficacy as low as 25%, the vaccination of calves would be of significant benefit.

The identification of immunological correlates of protection is essential to refine and prioritize vaccine candidates entering efficacy trials. IFN-γ has a central role in the context of BCG vaccination and protection against *M. bovis* [30], and vaccines which fail to prime for the production of antigen-specific IFN-γ responses show poor protection against virulent challenges [31,32]. In some studies, post-vaccination levels of IFN-γ correlate well with BCG-induced protection, although this is not always the case [31]. Although some studies indicate a non-specific release of IFN-γ in very young calves mediated by the activity of natural killer cells [33], this effect was not observed here in response to stimulation with PPD-B or with ESAT6 and CFP-10. We further demonstrate that antigen-specific IFN-γ induced by BCG correlated with protective efficacy when measured pre-*M. bovis* infection, but it was conversely associated with the extent of disease when measured post-*M. bovis* infection. This is in agreement with studies demonstrating that the frequency of central memory T lymphocytes expressing IFN-γ post-vaccination correlated significantly with protection [7,9], whereas the number of IFN-γ-expressing effector T cells present after *M. bovis* challenge was correlated with disease [7,34]. Post-challenge IFN-γ levels induced here via stimulation with the *M. bovis*-specific antigens ESAT-6 and CFP-10 were also correlated with the extent of bTB lesions, as previously reported [22].

The skin test data presented here is consistent with previous reports describing the compromised specificity of the tuberculin skin test in BCG-vaccinated animals. As many as 80% of calves vaccinated with BCG were reported to give skin test responses until at least 6 months after vaccination [35]. To overcome this, defined antigen combinations (antigens present in *M. bovis* but absent in BCG) have extensively been explored, and a DIVA skin test has been described that enables the differentiation of BCG-vaccinated individuals [5,20,36,37]. Field-testing of this DIVA skin test alongside BCG vaccination is now underway in the UK [38].

Our data add to the body of evidence showing that BCG vaccination of young calves can provide significant protection against infection with *M. bovis*. Strategic deployment of BCG vaccination in young animals, at a range of ages, could have a major impact on the incidence of bTB. This may be of particular relevance in settings where diagnostic test-and-slaughter is not feasible such as in LMICs, where *M. bovis* infection impacts both cattle and human populations significantly, as well as in countries such as the UK, where endemic bTB has significant economic implications.

## Figures and Tables

**Table 1:** Calves used in the study and the calculated doses of BCG and *M. bovis* used.

Animal	Treatment	Age at Vaccination	Sex	BCG Titre (×10^6^)	*M. bovis* Titre (×10^4^)
201540	BCG	1 day	Female	1.2	3.2
301548	BCG	1 day	Female	0.94	3.2
201554	BCG	1 day	Male	1.2	3.2
401633	BCG	1 day	Female	0.95	3.2
601642	BCG	1 day	Female	1.1	3.2
401549	Control	1 day	Male		3.2
301555	Control	1 day	Male		3.2
601558	Control	1 day	Female		3.2
501564	Control	1 day	Male		3.2
301583	BCG	3 weeks	Female	1.2	5.6
201582	BCG	3 weeks	Male	1.2	5.6
601600	BCG	3 weeks	Male	1.2	5.6
701601	BCG	3 weeks	Female	1.2	5.6
301604	BCG	3 weeks	Female	1.2	5.6
101623	Control	3 weeks	Female		5.6
201624	Control	3 weeks	Male		5.6
601628	Control	3 weeks	Female		5.6
701629	Control	3 weeks	Male		5.6

## Data Availability

Not applicable.
